# Wilson’s Disease in Childhood and the Challenges in Its Diagnosis: A Case Report

**DOI:** 10.7759/cureus.65847

**Published:** 2024-07-31

**Authors:** Vidhusree D, Krithika AP

**Affiliations:** 1 Pediatrics, Sree Balaji Medical College and Hospital, Chennai, IND

**Keywords:** kayser-fleischer ring, jaundice, dystonia, copper metabolism, wilson's disease

## Abstract

Wilson’s disease is a genetic neurometabolic disorder affecting copper metabolism in the body. It occurs due to mutations in the ATP7B gene. Here, we report a case of a 12-year-old boy, born out of a second-degree consanguineous marriage, who presented with complaints of jaundice for the past one year, poor scholastic performance, and behavioral abnormalities for the past one month. There was a history of multiple suicides in the maternal family, and liver disorder in the maternal uncle. Various examinations revealed jaundice, Kayser-Fleischer ring in eyes, and dystonia of the extremities with hepatosplenomegaly. Copper studies were inconclusive, and neuroimaging showed characteristic findings specific for Wilson’s disease. The child was treated with a low-copper diet, vitamin K, oral zinc acetate, oral D-penicillamine, trihexyphenidyl, baclofen, clonazepam, and propranolol.

## Introduction

Wilson’s disease is a genetic neurometabolic disorder inherited in an autosomal recessive manner, caused by mutations in the ATP7B gene on Chromosome 13q [1]. This mutation disrupts copper metabolism, leading to excessive copper accumulation primarily in liver and brain. The disorder was initially noted by Frerichs in 1861, who described a child with movement disorders and liver cirrhosis observed at autopsy [2]. However, a comprehensive clinical and pathological description was later provided by Wilson in 1912, calling it “progressive lenticular degeneration” [3]. In 1913, Rumpel found a relationship between excess copper and its effect on liver [4]. In 1929, Vogt, Haurowitz and Glazebrook identified that the patients dying of Wilson’s disease had excess of copper in the brain and liver [4]. In 1936, Policard et al. demonstrated excess copper in the cornea, or Kayser-Fleischer rings, in the affected patients [5].

More than 50 years after Wilson's landmark work, Wilson's disease was first documented in India by Wadia and Dastur in 1963 [1]. Since then, numerous studies and reports have emerged from dedicated Wilson's disease clinics throughout the country. Wilson's disease is globally prevalent, typically occurring in approximately 30-100/million people worldwide [6]. A study on hepatobiliary disorders in North India found that 7.6% of cases were attributed to Wilson's disease [7]. Although specific epidemiological data on neurological manifestations of Wilson’s disease in India are lacking, a specialty clinic in south India diagnoses 15 to 20 new cases annually [8].

## Case presentation

**Figure 1:**
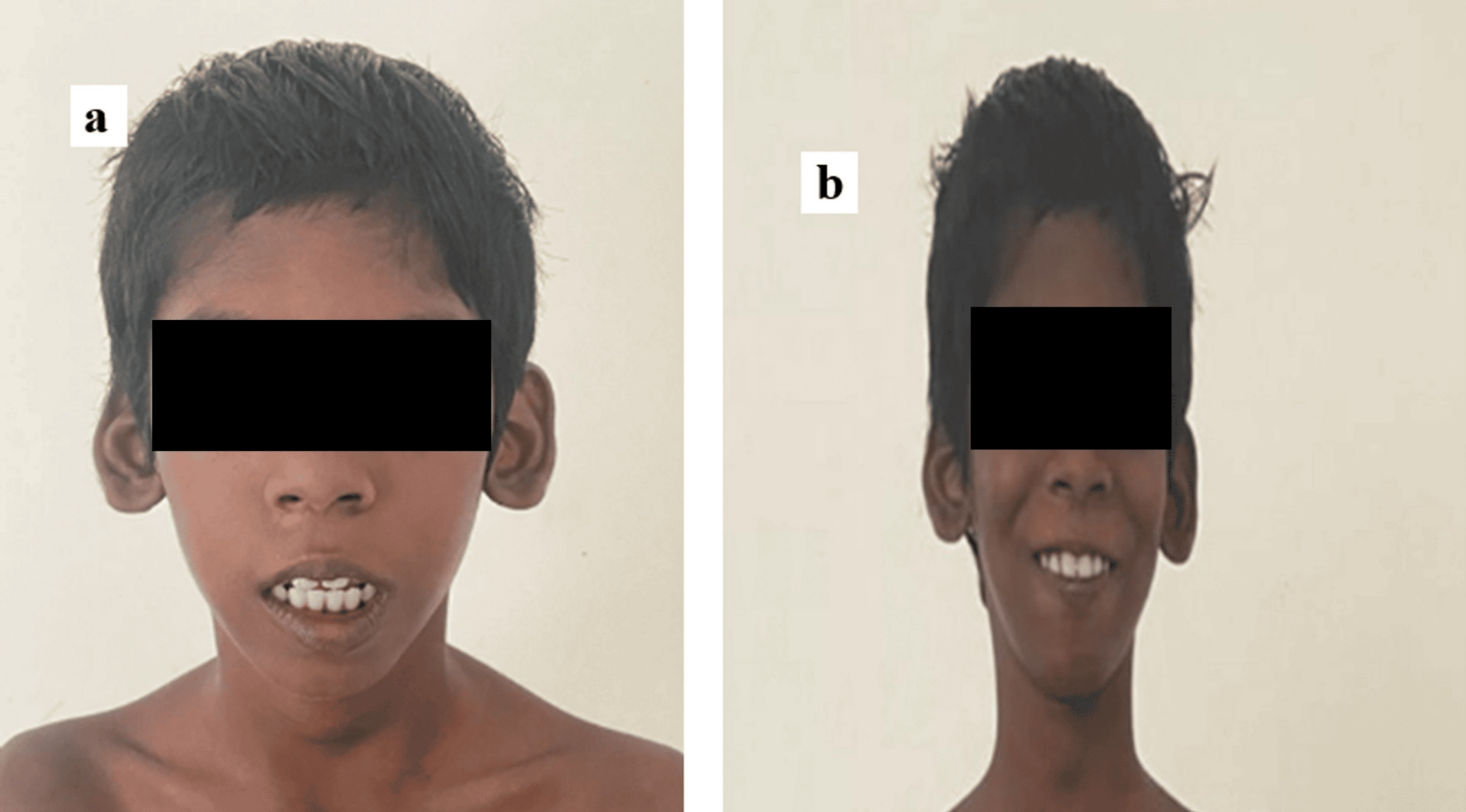
Clinical picture of the boy with dull demeanor (a) and a characteristic vacuous smile (b)

**Figure 2:**
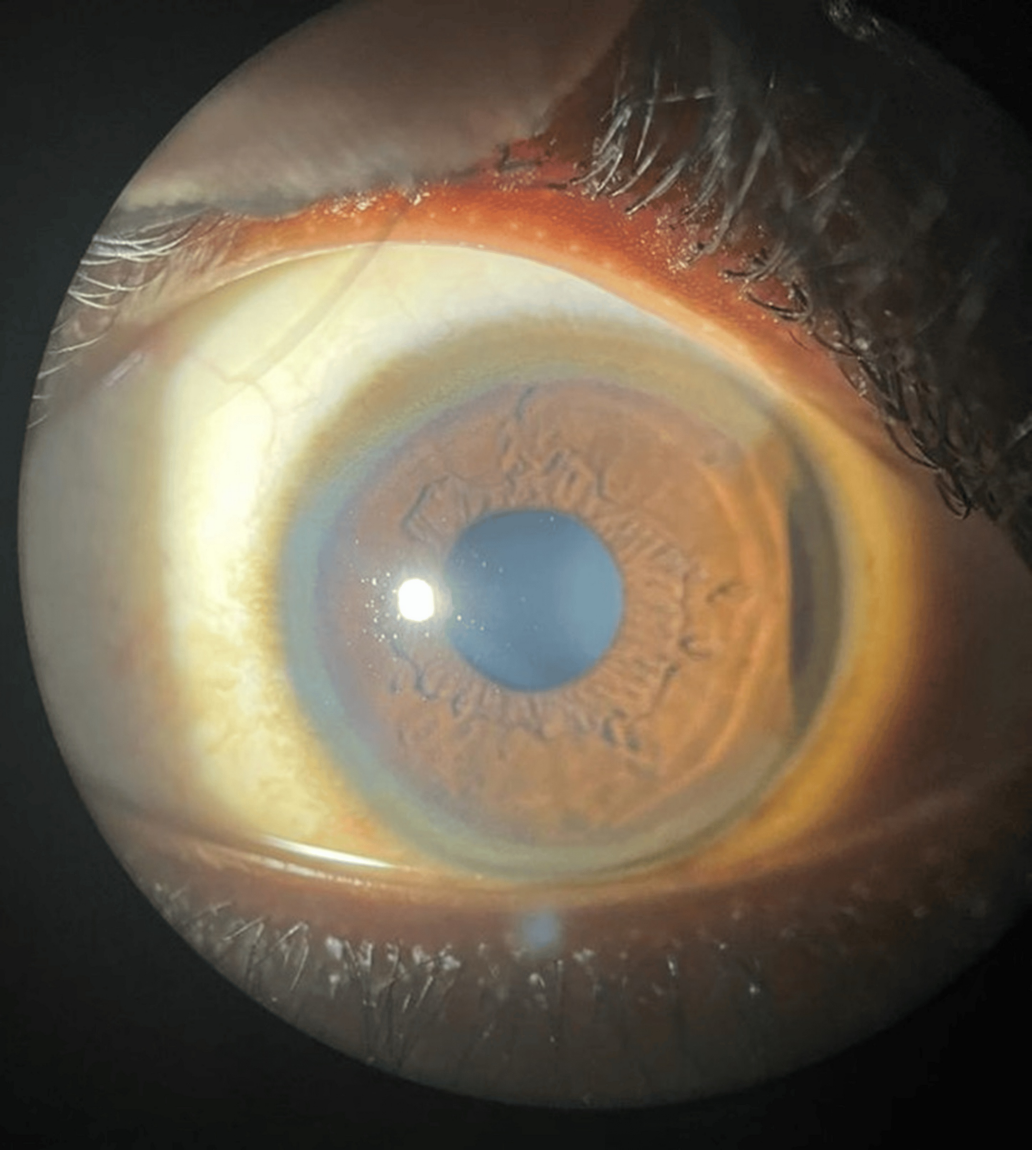
Clinical picture of the Kayser-Fleischer (KF) ring

**Figure 3:**
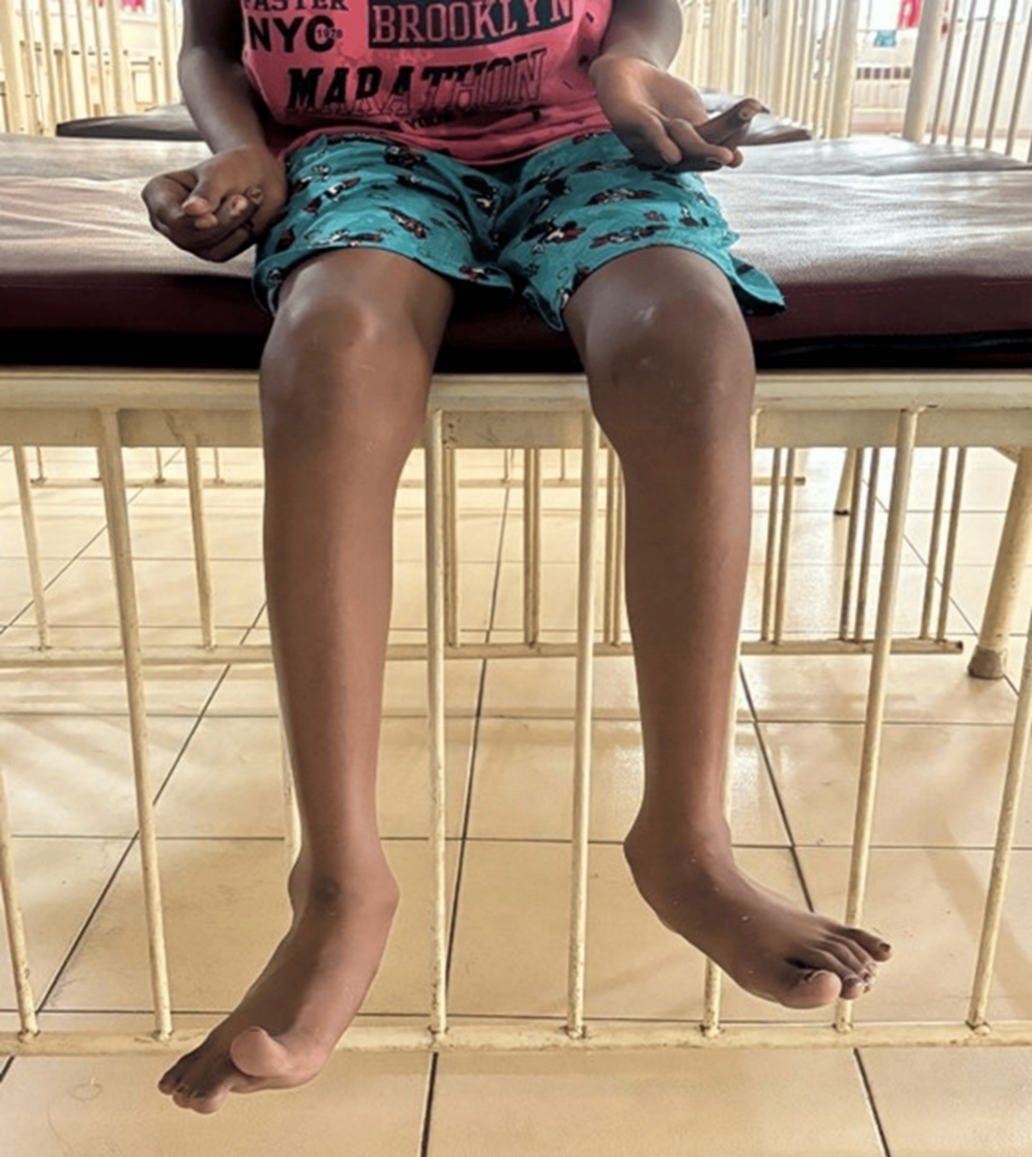
Clinical picture showing dystonia of all four limbs

A 12-year-old boy presented to our hospital with a clinical history of jaundice with dengue fever one year back; two months later he had another episode of jaundice and was treated with native medicine for three months. He presented with poor scholastic performance in the past one year; he had complaints of behavioral abnormalities, such as reduced interaction with peers and increased fights with his 15-year-old sibling, for the past one month, and a history of inappropriate laughter. He had a history of difficulty in speaking, specifically in pronouncing words, and decreased speed of speech for the past one month. He also had a history of stiffening and abnormal posturing of arms and feet with associated tremor while holding objects, for the past one month, which increased in severity in the past one week. He had a surgical history of circumcision done three months back. Developmental milestones were attained at appropriate ages. A family history of multiple suicides in the maternal family was noted. There was a history of recurrent jaundice in the maternal uncle, which was not evaluated properly. This boy was born out of a second-degree consanguineous marriage, with two first-trimester abortions noted in the mother. On examination, the patient appeared thin, poorly nourished and underweight. He had pallor and icterus. He displayed a dull demeanor with a characteristic vacuous smile (Figure 1). Notable clinical findings included Kayser-Fleischer (KF) ring in eyes (Figure 2), prominent ears, high arched palate and dystonia of the hands and feet (Figure 3).

He was categorized as stage I (prepubertal) under the Tanner staging of Sexual Maturity Rating (SMR) system. A neurological examination revealed dysarthria, impaired recent and remote memory, generalized hypertonia, and exaggerated deep tendon reflexes. Extrapyramidal signs were presented in the form of dystonia and coarse tremors, and cerebellar sign testing revealed incoordination. Hepatosplenomegaly was found on abdominal examination.

Laboratory investigations (Table 1) revealed anemia (Hb, 10.5 g/dL), thrombocytopenia (platelet count, 42,000/mm³), leukopenia (leucocyte count, 3960/mm³), hyperbilirubinemia (total, 2.1 mg/dL), elevated aspartate transferase levels, normal alanine transferase levels, hypoproteinemia, elevated prothrombin time (PT), and elevated activated partial thromboplastin time (APTT). Serum ceruloplasmin was elevated at 90 mg/dL, and the 24-hour urinary copper level was normal (19.0 µg/day). The D-penicillamine challenge test was done to assess 24-hour urinary copper excretion that was increased (1692.73 µg/24 hr). Copper studies were inconclusive.

**Table 1:** Laboratory results

Test parameters	Results	Normal range
Complete blood counts		
Hemoglobin (g/dL)	10.5	12.0–15.0
Total leucocyte count (cells/mm^3^)	3,960	4,500–13,500
Differential count (%)		
Neutrophils	61.2	25–57
Lymphocytes	30.1	35–65
Monocytes	6.6	2–10
Eosinophils	2.0	1–6
Basophils	1.0	<1
Platelets (cells/mm^3^)	42,000	1,50,000–3,50,000
Erythrocyte sedimentation rate (mm/hour)	22	0–20
Peripheral blood smear	Mild normocytic normochromic anemia with leukopenia and thrombocytopenia	
Renal function tests		
Urea (mg/dL)	12	12.84–42.80
Creatinine (mg/dL)	0.5	0.9–1.3
Uric acid (mg/dL)	1.5	4.4–7.6
Sodium (mEq/L)	139.5	136–145
Potassium (mEq/L)	3.37	3.5–5.1
Chloride (mEq/L)	110.6	96–106
Urine		
Albumin	Absent	Absent
Glucose	Absent	Absent
Bile salts	Absent	Absent
Bile pigments	Absent	Absent
Liver function tests		
Total bilirubin (mg/dL)	2.1	0.3–1.1
Direct bilirubin (mg/dL)	1.0	0.33–0.71
Indirect bilirubin (mg/dL)	1.1	0.2–0.9
Aspartate transaminase (IU/L)	96	0–35
Alanine transaminase (IU/L)	31	0–49
Alkaline phosphatase (IU/L)	233	54–369
Gamma glutamyl transferase (IU/L)	45	0–55
Total protein (g/dL)	6.2	6.0–7.8
Albumin (g/dL)	3.0	3.35–5.2
Globulin (g/dL)	3.2	2.5–3.5
Albumin:globulin (A:G) ratio	0.94	1.2:1–2:1
Coagulation studies		
Prothrombin time (PT) (s)	17.8	12.7–16.1
International normalized ratio (INR)	1.59	0.97–1.30
Activated partial thromboplastin time (APTT) (s)	43.6	33.9–46.1
Copper studies		
Serum ceruloplasmin (mg/dL)	90.0	23.6–41.2
24-hour urinary copper (mcg/day)	19.0	3.0–50.0
24-hour urinary copper after the D-penicillamine challenge test (mcg/day)	1692.73	3.0–50.0

**Figure 4:**
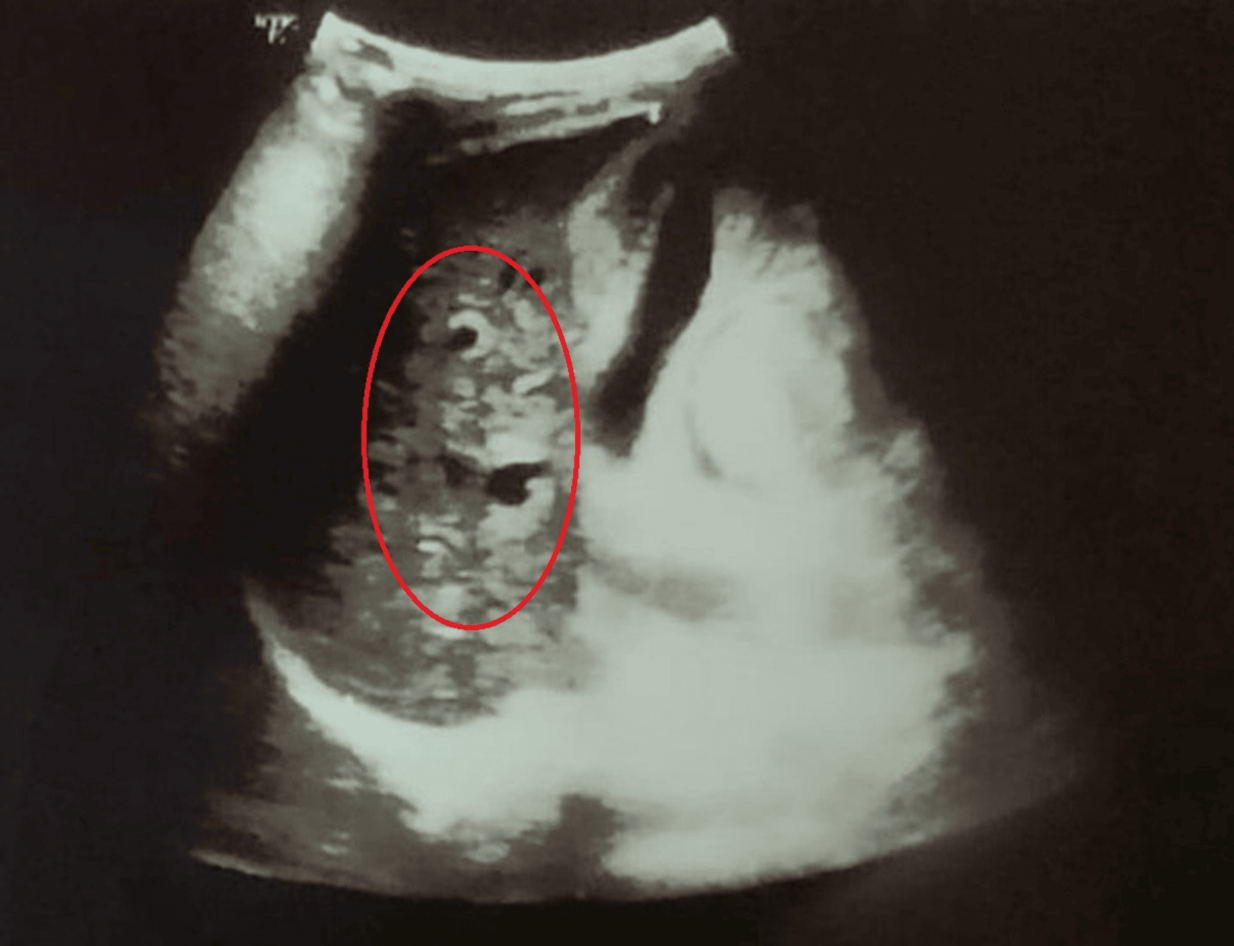
Abdominal ultrasound showing increased liver echotexture (red oval)

**Figure 5:**
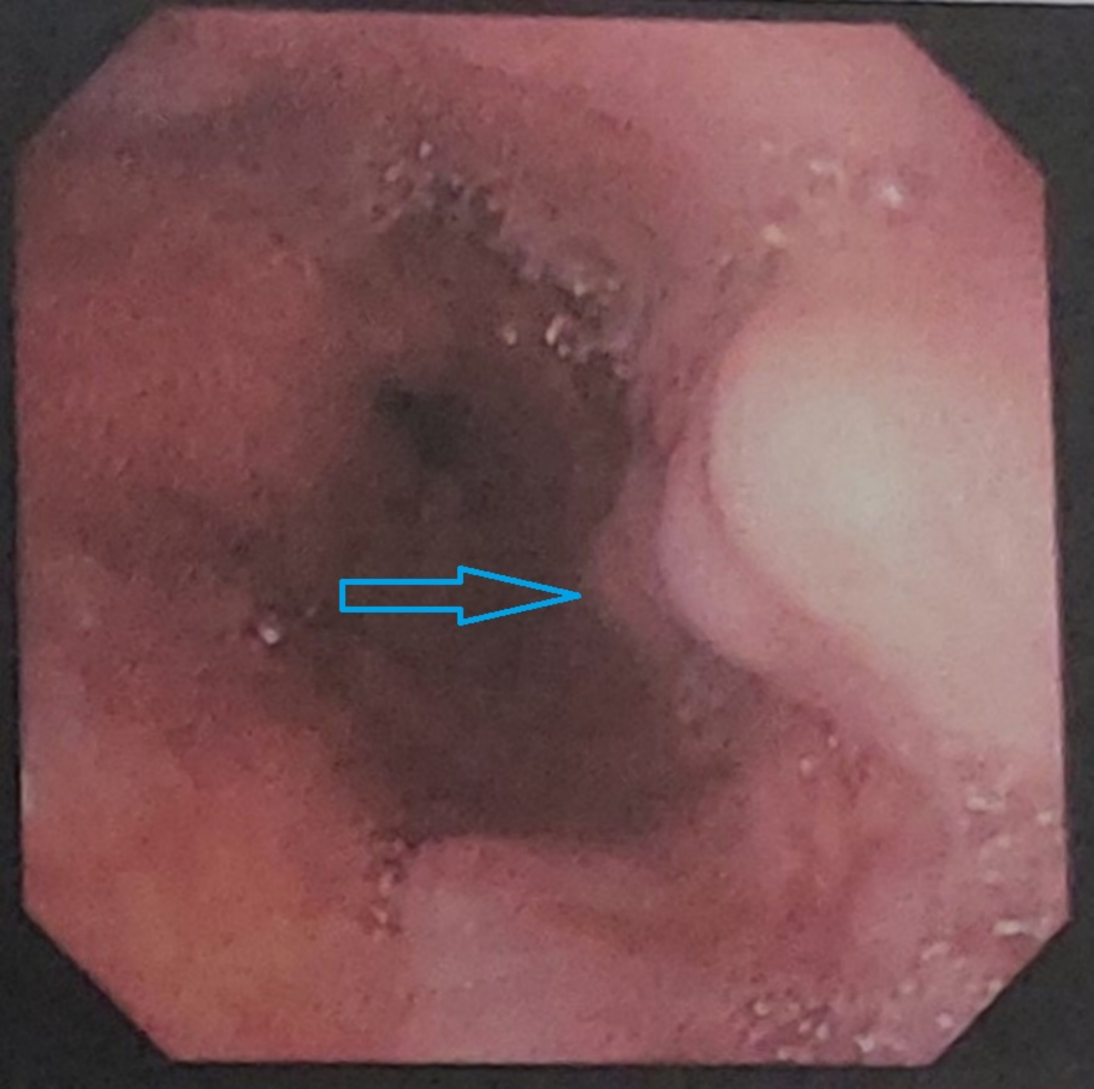
Upper-GI endoscopy showing grade II esophageal varices (blue arrow)

**Figure 6:**
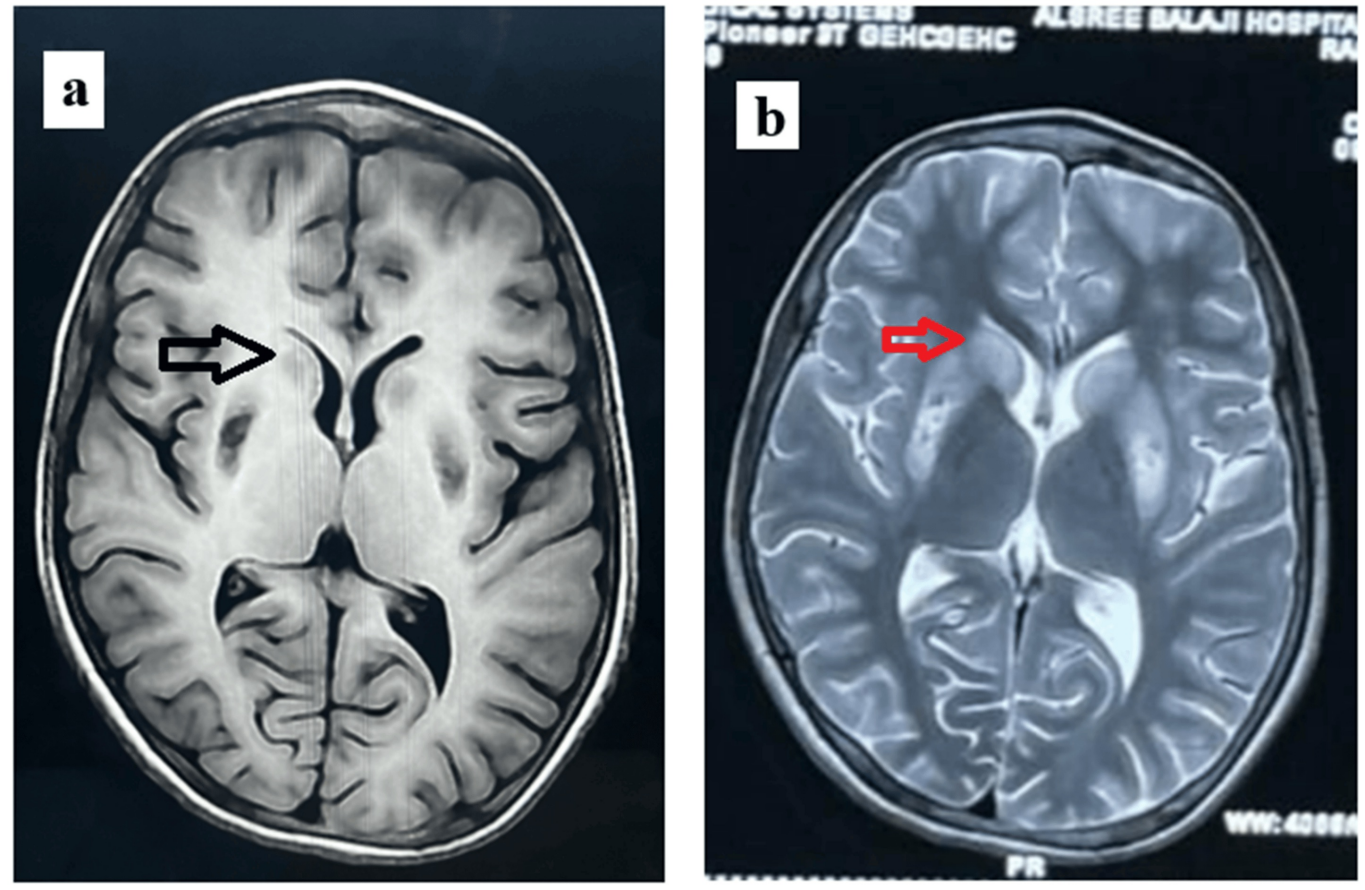
Brain MRI with T1-weighted sequences showing (a) hypointensity (black arrow) and T2-weighted sequences showing (b) hyperintensity (red arrow) of the bilateral putamen and caudate nucleus

**Figure 7:**
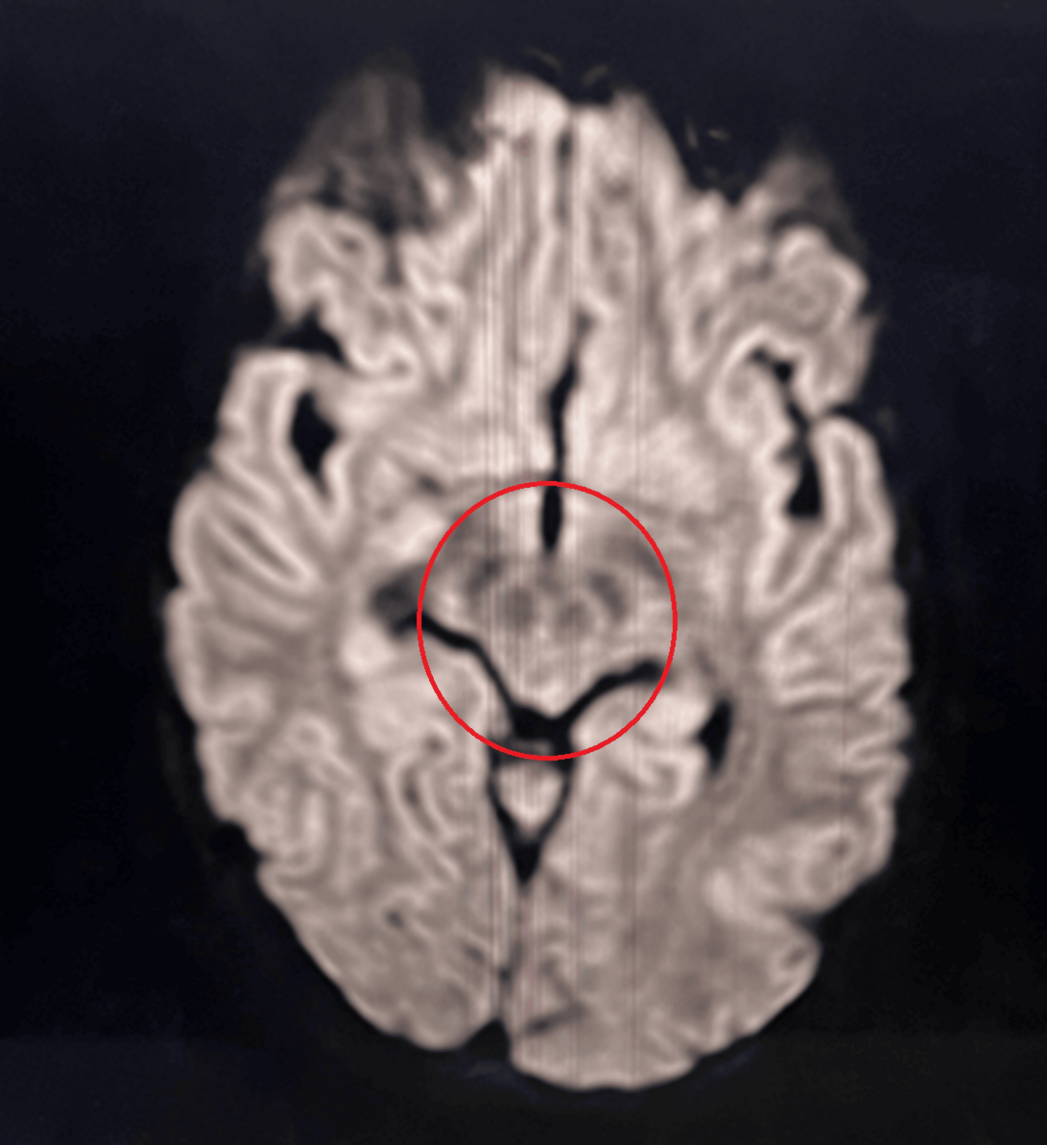
Diffusion-weighted imaging showing "face of the giant panda" sign (red circle)

Abdominal ultrasound showed hepatosplenomegaly and increased liver parenchymal echogenicity (Figure 4). Upper-GI endoscopy revealed grade II esophageal varices with mild portal hypertensive gastropathy (Figure 5). Brain MRI (Figure 6) demonstrated T2 hyperintensities and T1 hypointensities in the bilateral putamen and caudate nucleus, with diffusion-weighted imaging (Figure 7) showing the "face of the giant panda" sign.

The modified Leipzig score was used to diagnose Wilson’s disease with a cut-off of ≥4 being diagnostic of Wilson’s disease. Our patient scored 5, confirming the diagnosis of Wilson's disease (KF ring = 2, neurobehavioral symptoms = 2, MRI findings = 1).

His treatment included a low-copper-diet, vitamin K, oral zinc acetate, oral penicillamine, trihexyphenidyl, baclofen, clonazepam, and propranolol. Due to financial constraints, trientine was not administered, and D-penicillamine was used as the primary treatment. The pediatric hepatologist suggested liver transplantation within three months for reversal of symptoms. The patient is currently on the transplant waitlist.

## Discussion

In Wilson’s disease, mutations in the ATP7B gene impair copper incorporation into apo-ceruloplasmin and its excretion into the bile, leading to copper buildup in hepatocytes [9]. This results in lower ceruloplasmin levels, but by itself does not cause the disease. Excess copper inside hepatocytes keeps on binding to metallothionein, but eventually overwhelms them, causing hepatocyte damage through oxidative stress, mitochondrial dysfunction and inhibited protein synthesis. Damaged hepatocytes release copper into the blood, increasing free serum copper levels. This leads to copper deposition and toxicity in the brain and other tissues, and hence also known as hepatolenticular degeneration. Urinary excretion is unable to fully compensate for decreased biliary excretion. Liver involvement is nearly universal in Wilson’s disease, affecting almost all cases, clinically or biochemically, often starting in childhood. Symptoms vary from asymptomatic to overt liver cirrhosis [10]. Acute hepatitis or fulminant hepatic involvement occurs in 10% of cases, with chronic liver issues in one-third of the cases. Rarely, long-standing Wilson’s disease may lead to hepatocellular carcinoma or cholangiocarcinoma.

In Indian cases of Wilson’s disease, most common neurological symptoms are of extrapyramidal manifestations [11]. Parkinsonism is found in nearly two-thirds of patients, while dystonia and ataxia affect about one-third. Other manifestations include pyramidal features in 16% and less frequent occurrences of chorea, athetosis, and myoclonus. Craniofacial dystonia can lead to characteristic facial expressions like a "vacuous smile" and drooling. Seizures are seen in 8%-38% of cases, possibly due to copper toxicity, liver-related issues or pyridoxine deficiency from penicillamine use. Cognitive deficits are prevalent, affecting motor speed, verbal working memory, and attention in many cases. Neurological involvement in Wilson’s disease is diverse and often includes KF rings in nearly all affected patients. Symptoms can range from being subtle to rapidly progressive disease, resembling hepatic encephalopathy in those with cirrhosis. Dysarthria is a common feature seen in up to 97% of cases, with various types like ataxic and hypophonic speech. Cerebellar ataxia is seen in 30%-75% of cases, affects voluntary force adjustments and can lead to movement coordination issues. Dystonia is reported in 38%-69% of patients; it ranges from mild to severe and can involve focal areas like the face or limbs. Tremors are seen in up to 55% of cases, and may be postural or intentional, resembling wing-beating or essential tremors. Parkinsonism is seen in 12%-58% of cases, and manifests as bradykinesia and rigidity. Chorea and athetosis involve involuntary limb movements, while cognitive impairment and psychiatric symptoms are common. Seizures, dysautonomia, and other manifestations like hyperreflexia or myoclonus may also occur.

Ocular involvement in Wilson’s disease includes KF rings, which are golden-brownish rings caused by copper deposits in the Descemet’s membrane on the cornea's periphery [9]. They are present in nearly all patients with neurological symptoms and about half of those with liver issues. KF rings are typically detected using a slit-lamp examination or anterior segment optical tomography, which can also measure their size. Chelation therapy, zinc treatment, or liver transplantation can reduce or eliminate KF rings over time. Additionally, sunflower cataracts, caused by copper accumulation in the lens, can be identified through a slit-lamp examination.

Other system involvements include osseomuscular features such as large-joint polyarthritis, osteomalacia, refractory rickets, spontaneous fracture and limb deformities. Renal features include nephrolithiasis, vitamin-D-resistant rickets, and renal tubular acidosis. Cardiac features include cardiomyopathy and cardiac arrythmias. Autonomic dysfunction can also be seen, such as sleep disturbances and generalized hyperpigmentation. It can also cause Coombs-negative hemolytic anemia.

Biochemical findings specific for Wilson’s disease are reduced to normal serum ceruloplasmin levels (normal range, 20-40 mg/dL), elevated 24-hour urinary copper (>100 mcg/dL), and reduced serum copper levels. The most accurate test with high sensitivity is that for hepatic copper (>250 mcg/g) [12]. In neuroimaging, brain CT can show cortical atrophy and ventricular dilatation. MRI can show “face of the giant panda” in midbrain and “face of the miniature panda” in pons. T2 hyperintensities are seen in the bilateral basal ganglia, thalamus, midbrain, cerebrum and brainstem, and T1 shows hypointensities in the basal ganglia, thalamus and midbrain [13].

The Leipzig scoring system for the diagnosis of Wilson's disease (Table 2) was developed in 2001 at the Eighth International Meeting on Wilson’s Disease [14]. This scoring system had limitations in relation to diagnosis in very young children [15]. Hence, this scoring system was modified with a few additions and was validated in 70 patients of Wilson’s disease (Table 3) [9]. The additional points in the modified scoring system gave more importance to very low serum ceruloplasmin levels of ≤5 mg/dL as compared to moderately low levels of >5 mg/dL; liver copper estimation and D-penicillamine challenge test were excluded.

**Table 2:** Original Leipzig scoring system for Wilson's disease ^*^Upper limit of normal (ULN) for 24-hour urinary copper is 50 µg/24 hr.

Features	Score
Kayser–Fleischer corneal rings	Present = 2
	Absent = 0
Serum ceruloplasmin	<10 mg/dL = 2
	10-20 mg/dL = 1
	Normal (>20 mg/dL) = 0
24‑hour urinary copper (in the absence of acute hepatitis)^*^	>2x ULN = 2
	1x to 2x ULN = 1
	Normal = 0
	Normal, but >5x ULN after D-penicillamine = 2
Coombs-negative hemolytic anemia with liver disorder	Present = 1
	Absent = 0
Genetic mutation	Detected on both chromosomes = 4
	Detected on one chromosome = 1
	Not detected/test not done = 0
Liver copper (in the absence of cholestasis)	Liver copper estimation
	>250 μg/g = 2
	50–250 μg/g = 1
	Normal (<50 μg/g) = (-1)
	Rhodamine‑positive hepatocytes = 1
Neurobehavioral symptoms or typical features on brain MRI	Present = 2
	Absent = 0
Total score	≥4: diagnosis highly likely
	2–3: diagnosis probable, more tests needed
	0–1: diagnosis very unlikely

**Table 3:** Modified Leipzig scoring system for Wilson's disease

Features	Score
Kayser–Fleischer corneal rings	Present = 2
	Absent = 0
Serum ceruloplasmin	0–5 mg/dL = 3
	6–11 mg/dL = 2
	12–20 mg/dL = 1
	Normal (>20 mg/dL) = 0
24‑hour urinary copper (in the absence of acute hepatitis)	>100 mcg/day = 2
	40–100 mcg/day = 1
	<40 mcg/day = 0
Coombs-negative hemolytic anemia with liver disorder	Present = 1
	Absent = 0
Genetic mutation	Detected on both chromosomes = 4
	Detected on one chromosome = 1
	Not detected/test not done = 0
Liver biopsy	Orcein‑positive or rhodamine‑positive granules = 1
Neurobehavioral symptoms	Present = 2
	Absent = 0
Typical features suggestive of Wilson’s disease on brain MRI	Present = 1
	Absent = 0
Family history of Wilson’s disease	Sibling death from liver or neurological features suggestive of Wilson’s disease = 1
Total score	≥4: definitive diagnosis of Wilson’s disease
	3: possible Wilson’s disease and further evaluation needed
	≤2: Wilson’s disease unlikely

In our patient, the modified-Leipzig score was 5 (Kayser-Fleischer corneal rings = 2, neurobehavioral symptoms = 2, brain MRI findings suggestive of Wilson’s disease = 1). Therefore, even when the copper studies were inconclusive, this scoring system was used for establishing the diagnosis of Wilson’s disease. This case is an example of the significance of clinical diagnosis over laboratory diagnosis.

In Wilson’s disease, family screening is important as siblings have a 25% risk of Wilson’s disease, while offspring and parents have 0.5% risk. In the case of absent genetic testing, siblings, first-degree relatives, parents, and offspring should undergo an assessment for KF rings along with serum ceruloplasmin and 24-hour urinary copper examinations. Even if diagnosis of Wilson's disease is ruled out during the first screening, they should be monitored every 6 to 12 months. The sibling of our patient, who was 15 years of age, was screened negative.

It was found that up to 90% of the Indian neurological Wilson's disease patients can recover back to premorbid functionality with adequate therapy and follow-up [11]. Wilson’s disease patients with hepatic presentation carry a five times higher risk of mortality than those with neurological presentation. Patients with acute hepatic involvement with hepatic encephalopathy have more than 50% mortality.

The recommended treatment of Wilson’s disease is avoidance of copper-rich foods. The dietary copper intake is restricted to less than 1 mg/day. This child was provided with a specifically curated diet chart. Copper-chelating drugs such as D-penicillamine, trientine, and unithiol are used. For neurological symptoms such as dystonia and tremors, symptomatic therapy can be given with anticholinergics and benzodiazepines. Antipsychotics can be given to manage behavioral abnormalities. Lesional surgery and deep brain stimulation (DBS) are also being used in the treatment of Wilson’s disease. They target ventral nucleus of the thalamus and globus pallidus internus, treating tremor, dystonia and drug-refractory extrapyramidal features. If the child develops decompensated liver failure, then the definitive treatment is liver transplantation.

## Conclusions

This case highlights the clinical spectrum and diagnostic challenges of Wilson's disease in childhood, emphasizing the importance of a multidisciplinary approach in managing this genetic disorder with both hepatic and neurological involvement. This case emphasizes the importance of evaluating an adolescent child with behavioral changes rather than dismissing it. This case also emphasizes the importance of a good clinical history, as there was a family history of neurological and hepatic conditions that were undiagnosed in this case, and the importance of screening the sibling. Early recognition and adequate treatment can improve the long-term outcome in such patients. A pan-India registry of Wilson's disease cases is required to help increase awareness and aid in the standardization of the management protocol.
